# Religion and professional experience: Are they predictors of nurses’ spiritual intelligence? Cross-sectional study

**DOI:** 10.1590/0034-7167-2024-0217

**Published:** 2024-12-16

**Authors:** Jhon Alex Zeladita-Huaman, Juana Matilde Cuba-Sancho, Martha Brigida Martina-Chávez, Roberto Zegarra-Chapoñan, Henry Castillo-Parra

**Affiliations:** I Universidad Nacional Mayor de San Marcos. Lima, Peru; II Universidad María Auxiliadora. Lima, Peru; III Universidad San Buenaventura. Medellín, Colombia

**Keywords:** Intelligence, Religion, Spirituality, Nurses, Daily Activities., Inteligência, Religião, Espiritualidade, Enfermeiras e Enfermeiros, Atividades Cotidianas.

## Abstract

**Objectives:**

to analyze the relationship between religion and professional experience with spiritual intelligence in nurses

**Methods:**

cross-sectional and analytical study carried out in 2021, with the participation of 544 nursing professionals working in health facilities in Peru during the COVID-19 pandemic. Multiple regression analysis and Pearson’s correlation were used to analyze the data.

**Results:**

in nurses, a healthy level of spiritual intelligence predominated (42.8%). Those who did not profess a religion were more likely to have a lower spiritual intelligence score (global scale and dimensions); however, experienced nurses were more likely to have higher spiritual intelligence (global scale and dimensions) than novice nurses (p<0.05).

**Conclusions:**

spiritual intelligence in nurses was predicted by religion and professional experience. This finding suggests that spiritual intelligence in nursing is consolidated through religious practices and during professional practice.

## RESUMO

**Objetivos:**

analisar a relação entre religião e experiência profissional com a inteligência espiritual em enfermeiros.

**Métodos:**

estudo transversal e analítico realizado em 2021, com a participação de 544 profissionais de enfermagem que trabalhavam em estabelecimentos de saúde no Peru durante a pandemia de COVID-19. Para a análise dos dados, foram utilizados a análise de regressão múltipla e a correlação de Pearson.

**Resultados:**

nos enfermeiros, predominou um nível saudável de inteligência espiritual (42,8%). Aqueles que não professavam uma religião tinham maior probabilidade de apresentar uma pontuação menor de inteligência espiritual (escala global e dimensões); no entanto, os enfermeiros experientes tiveram maior probabilidade de apresentar maior inteligência espiritual (escala global e dimensões) do que os enfermeiros novatos (p<0,05).

**Conclusões:**

a inteligência espiritual nos enfermeiros foi predita pela religião e pela experiência profissional. Este achado sugere que a inteligência espiritual na enfermagem se consolida por meio de práticas religiosas e durante o exercício profissional.

## INTRODUCTION

Spiritual intelligence (SI) has a polysemic and varied meaning. However, it refers to a person’s ability to search for a deep meaning to their existence^(1)^; in addition, to the effort to seek a purpose in their daily and professional life in order to understand the meaning^(2)^ and satisfaction of life. It includes the highest level of growth in the cognitive, ethical, emotional and interpersonal areas, which allow the individual to fully achieve internal and external integrity^(3)^. Based on these understandings, scales have been validated to measure SI during everyday life^(2)^ and in the care practice of healthcare personnel^(4)^. Furthermore, taking into account that SI is associated with the development and transfer of knowledge, continuous learning, self-control and work performance^(5)^. This intelligence is a prerequisite and necessary component of leadership to achieve sustainable development and is aligned with the Sustainable Development Goals^(6)^.

In the scientific community, there has been much discussion about the association between SI and religion. Initially, when defining SI, it was postulated that this ability would not necessarily have a connection with religion, since being religious does not imply having greater SI; but this intelligence could be expressed through religion^(7)^. However, recent evidence has opened up the debate on this discrepancy. In this sense, a study carried out in the Kingdom of Saudi Arabia found that students from religious schools scored higher in EI; in addition, using a multivariate analysis, it reported that religiosity predicts SI^(8)^. Similarly, another study of nurses in China concluded that religious beliefs are a predictor of SI^(9)^.

A secondary study found that nurses with high SI have greater work competence, a strengthened personal sense of self-care, better moral performance, personal excellence and flexibility; aspects that contribute to increasing the quality of nursing care^(10)^. While another systematic review reported that nurses’ education, spirituality or religious beliefs, age, years of clinical experience and workplace influence nurses’ understanding of spirituality and spiritual care^(11)^. However, no studies were found that analyzed the relationship between religious practices and work experience with SI in Latin American nurses, despite the fact that, in their daily work, they offer spiritual care as a useful strategy to ensure comprehensive care, with a vocation of service and respect for the patient’s religious beliefs^(12)^. Furthermore, in order to understand the spiritual needs of others, it is necessary to understand one’s own spirituality, values, beliefs^(9)^, symbols, images and imaginaries that describe the way they live, in other words, their daily lives^(13)^.

Studies conducted prior to the COVID-19 pandemic have reported that nurses have a moderate level of SI^(5)^; while research conducted during the pandemic has highlighted an acceptable level^(14)^. On the other hand, it has been reported that this intelligence is associated with sociodemographic and epidemiological characteristics, such as age, marital status, religion, professional experience and the history of having been infected by COVID-19^(14,15)^; as well as factors related to their work environment, such as communication, perceived self-efficacy at work^(14)^, work performance^(5)^, the art of nursing, their clinical skills, attributes of practice and personal commitment^(16)^.

Research into SI in nurses and its individual predictors has implications for understanding the influence that personal characteristics have on SI during daily work in nursing, an aspect that becomes relevant because this ability favors their adaptation to the work environment and improves the quality of care they offer to patients^(17)^. In addition, it is an essential starting point to promote the development of interventions that increase SI, which would facilitate improved work performance^(18)^ and increase organizational efficiency^(19)^. It also makes it possible to reflect on the importance of spiritual care in formal patient care. In this respect, there has been insufficient research exploring the association between SI and work-related variables, such as professional experience, sociodemographic characteristics and the practice of a religion^(5,9)^.

## OBJECTIVES

To analyze the relationship between religion and professional experience with SI in nurses.

## METHODS

### Ethical aspects

This research was approved by the Research Ethics Committee of the Maria Auxiliadora University and all the participants gave their free and informed consent, which was obtained virtually. As the data was collected using an electronic form, one of the study researchers assigned an identification code to the information collected to guarantee anonymity and protect the identity of the participants before statistical analysis. The file containing the e-mail, the only sensitive data collected, was then stored in a separate file from the database on a private, password-protected server.

### Design, study site and period

This is a quantitative, analytical, cross-sectional study carried out in the city of Lima, Peru, between April and July 2021. The report was oriented according to the STROBE.

### Population and sample: inclusion and exclusion criteria

Nursing professionals who worked in public and private healthcare establishments and who had an electronic device (laptop or cell phone) with internet access to access the virtual form were included. Nursing professionals who were not resident in Peru were excluded, as were those who worked exclusively in the administrative and/or teaching fields. To determine the sample size, we considered the detection of small regression effects (R^2^=0.02) with a statistical power of at least 80% and a type I error probability of 5%. Under these criteria, a minimum sample of 305 subjects was established. However, as a non-probability convenience sampling method was used, in order to increase statistical power and reduce the impact of individual biases, the sample was increased to 554 participants. In addition, we sought to diversify the sample by inviting nursing professionals who worked in public and private sector healthcare establishments in different regions of Peru.

### Study protocol

The technique used was a virtual survey and the instrument was a self-administered questionnaire developed in Google Forms. Data was collected through social networks, such as WhatsApp groups of nursing specialization students from Peruvian universities and Facebook groups of organizations that bring together nursing professionals both at union level and in hospital institutions. In addition, direct invitations were sent by email to nursing professionals. The possible predictors of SI considered were: gender, professional experience (length of experience in years since graduation), type of work establishment, whether they did remote work, service in which they worked, and whether they had been diagnosed with COVID-19. For the religious affiliation variable, two categories were considered: religious (if the participant defined themselves as Catholic, Evangelical, Adventist or another type of religion) and non-religious (when the participant considered themselves agnostic or reported that they did not practice any religion).

For the SI variable, we used the SI in health practice scale, made up of 18 items^(4)^, grouped into three dimensions: spiritual experience in practice, existential thinking and transcendental awareness. The response options ranged from 1 to 4 points (1 = not at all true for me, 2 = somewhat true for me, 3 = very true for me and 4 = totally true for me). The levels were: unhealthy (<45 points), improving (45-58 points) and healthy (>58 points).

This scale was initially designed to measure SI in Peruvian health professionals and, through a psychometric study with exploratory factor analysis, it was determined that it has adequate construct validity and reliability (Cronbach’s alpha of 0.90)^(4)^. It was subsequently used in another study carried out with the Peruvian population, in which it was reaffirmed that the scale has adequate reliability (Cronbach’s alpha of 0.82)^(20)^.

For this study, the SI scale was validated through expert evaluation, with the participation of three nurses specializing in mental health and a doctor with experience in SI research. The evaluation was carried out according to the content validity index (CVCic)^(21)^, and adequate agreement was obtained between the pertinence criterion (CVCic = 0.944) and the clarity criterion (CVCic = 0.935). In addition, with the data collected, it was determined that the scale has high reliability (McDonald’s Omega coefficient of 0.93).

The probability of bias was controlled by using validated and reliable instruments, with clear precision of the objectives and encouraging participants to answer the questionnaire; minimizing losses due to non-response and, above all, guaranteeing the anonymity of the participants, given that this was a very sensitive subject.

### Analysis of results and statistics

Based on the selected variables, such as religiosity, predictors and SI, descriptive and inferential statistics were used as appropriate. The assumption of normality of the continuous measurement variables was checked using the Shapiro-Wilk test, to confirm the use of parametric tests. Pearson’s correlations between each of the dimensions of SI were then determined. Finally, four regression models were estimated to predict the SI score and each of its dimensions, based on variables such as gender, length of experience and religion. For the resulting models, the assumptions of multicollinearity, homogeneity of variance and normality were reviewed. Graphical and statistical tools were used to check that these assumptions were met, such as quantile-quantile graphs, a significance value greater than 0.05 in the Shapiro-Wilk test, Levene’s test and values less than 10 in the variance inflation factor (VIF). In this way, all the assumptions were satisfactorily met. Likewise, the existence of atypical cases was explored; however, no case was considered problematic (>1) according to the Cook’s D criterion. The level of statistical significance was set at 0.05 as the cut-off point, according to the conventions used in the existing literature. All the analyses were carried out using the R v 4.1.0 software.

## RESULTS

A total of 544 nurses from 19 cities in Peru took part. The majority were women (83.1%), with 10 years or less experience (65.8%), working in a hospital or clinic (78.1%), not doing remote work (86.8%) and working in Emergency and Inpatient services (61.0%) (Table 1). The average age was 37.36 ± 10.15 years. As for religious affiliation, 77.9% (424) said they were Catholic, 13.1% (71) were Evangelical, 0.7% (4) Adventist, 5.7% (31) followed another religion and 2.6% (14) considered themselves agnostic or professed no religion.

**Table 1:** Characteristics of the nurses interviewed, 2021

Característica	n	%
Sex		
Male	92	16.9
Female	452	83.1
Professional experience (years)		
Under 5	210	38.6
From 5 to 10	148	27.2
From 11 to 15	80	14.7
From 16 to 20	36	6.6
Over 20	70	12.9
Type of workplace		
Hospital or clinic	425	78.1
Health centers	88	16.2
Other care center^*^	31	5.7
Does remote work		
No	472	86.8
Yes	72	13.2
You have been diagnosed with COVID-19		
No	314	57.7
Yes	230	42.3
Service		
Emergency	212	38.9
Hospitalization	120	22.1
Primary care	76	13.9
Intensive care unit	55	10.2
Other service†	81	14.9

* *Includes private clinics, specialized care centers, among others; †Includes pediatric and oncology services, among others.*

### Spiritual intelligence

According to the data analyzed, 42.8% (233) of the participants reported a healthy SI; 41.4% (225) showed an improving SI and 15.8% (86) showed an unhealthy SI. Table 2 describes the scores obtained on the SI scale and its dimensions.

**Table 2:** Average score, standard deviation, maximum and minimum of the spiritual intelligence scale and its dimensions in nurses, 2021

	Mean ± SD	Minimum - Maximum
Dimension 1: Spiritual experience in practice	19.92 ± 3.38	10 - 24
Dimension 2: Existential thinking	19.80 ± 4.10	7 - 28
Dimension 3: Transcendental consciousness	15.10 ± 2.87	5 - 20
Total spiritual intelligence	54.82 ± 9.54	28 - 72

In all the indicators of the dimension Spiritual experience in practice, which refers to the behavioral coherence of SI, the category “Totally true for me” predominates, except in one indicator; in addition, in three of these, the percentage is higher than 50%. In almost all the indicators of the other two dimensions, the category “Quite true for me” predominates (Table 3).

**Table 3:** Percentage of indicators according to dimensions of spiritual intelligence in nurses, 2021

Indicators	Nothing real for me	Something real for me	Very true for me	Totally true for me
	%	%	%	%
Dimension: Spiritual experience in practice				
2. No matter the place or circumstance, I always act according to my principles.	0.7	9.7	34.6	55.0
5. When I am dedicated to the noble mission of my nursing practice, my strength multiplies.	1.5	14.7	33.3	50.6
8. I believe that taking care of my body and the bodies of patients is a sacred duty.	0.6	15.6	30.0	53.9
11. When a patient needs me, I always find the time to help.	0.4	12.1	40.1	47.4
14. Beyond the human plane, there is a higher being with whom we can relate.	2.4	17.6	41.7	38.2
17. I'm sure that helping others or showing solidarity with patients is my mission in life.	1.1	17.3	33.8	47.8
Dimension: Existential thinking				
1. I believe that everything in life has a profound meaning.	2.6	24.3	38.4	34.7
4. When I think of the miracle of my existence, I am filled with joy.	5.0	17.8	32.0	45.2
7. My mind calms down when I reflect on a spiritual text.	5.5	25.2	41.4	27.9
10. I often reflect on the meaning of events in my life.	1.5	24.1	49.6	24.8
13. I am able to reflect deeply on what there may be beyond death.	7.9	31.8	39.0	21.3
16. I am aware that there is a deeper connection between other people and me.	8.1	27.6	33.8	30.5
Dimension 3: Transcendental consciousness				
18. It's hard for me to think of anything beyond the physical and material world.	32.9	35.7	23.0	8.5
3. My moments of spiritual practice renew my physical strength.	2.6	19.1	37.1	41.2
6. In my free time, I like to enjoy nature, such as a garden, park or rooftop.	6.6	34.0	33.8	25.6
9. When I experience failure, I can still find meaning in it.	2.0	29.2	46.9	21.9
12. I define myself by my deepest self, not by my physical self.	0.7	18.4	49.3	31.6
15. I often see situations and options more clearly when I meditate, pray.	2.4	18.0	42.8	36.8

### Predictors of spiritual intelligence

The first multiple regression analysis model to determine the predictors of SI reported that nurses with 16 and 20 years of professional experience had 4.42 points more SI; in addition, those with more than 20 years of professional experience had a higher SI score than participants with less than five years of experience (p < 0.05). Finally, nurses who did not consider themselves religious had 9.65 fewer SI points than those who said they professed some religion (p < 0.001). The proposed model explains 5% of the variability in SI (Table 4).

**Table 4:** Regression models for predicting spiritual intelligence and its dimensions, 2021

	Spiritual intelligence		Spiritual living in practice		Existential thinking		Transcendental consequence	
	*b*	*B*	*b*	*B*	*b*	*B*	*b*	*B*
Intercept	54.78^*^	-	19.86^*^	-	20.11^*^	-	14.80^*^	-
Sex (female)	-0.91	-0.04	-0.39	-0.04	-0.55	-0.05	0.03	0.00
Work experience (5 to 10 years)	0.39	0.02	0.15	0.02	0.20	0.02	0.04	0.01
Work experience (11 to 15 years)	2.11	0.08	1.31†	0.14	0.21	0.02	0.58	0.07
Work experience (16 to 20 years)	4.42†	0.12	1.49‡	0.11	1.60‡	0.10	1.34†	0.12
Professional experience (over 20)	2.58‡	0.09	0.98‡	0.10	0.40	0.03	1.21^**^	0.14
Profess a religion (non-religious)	-9.65^*^	-0.16	-2.91†	-0.14	-3.80^*^	-0.15	-2.93^*^	-0.16
R^2^	0.05^*^		0.05^*^		0.03†		0.06^*^	

b - Non-standardized coefficient; B - Standardized coefficient;

* p value < 0.001; †p < 0.01; ‡p value < 0.05

The other three multiple regression analysis models to determine the predictors of the dimensions of SI, presented in Table 4, show that religious affiliation and professional experience were predictors in all dimensions. With regard to the latter predictor, the score for the spiritual experience in practice dimension was significantly predicted by the three ranges of professional experience considered in this study. As for the existential thinking dimension, there were differences only between participants with less than five years’ professional experience and those with between 16 and 20 years’ experience, with the latter scoring 1.60 points higher in this dimension (p < 0.05). Finally, in the transcendental awareness dimension, nurses with between 16 and 20 years’ experience scored 1.34 points higher (p < 0.01), and those with more than 20 years’ experience scored 1.21 points higher (p < 0.01) compared to those with less than five years’ experience.

### Post-hoc statistical power analysis

Among the models analyzed, the one with the smallest effect size was the existential thinking prediction model, which, according to the R^2^ reported, had a small effect (R^2^ = 0.03), equivalent to an f^2^ equal to 0.03. The value for a model with 3 predictors, an α error of 0.05 and a total sample size of 544 people resulted in a statistical power of 0.95. This power, being higher than the minimum of 0.80, indicates that the study had a sufficient sample size for the analyses presented.

## DISCUSSION

In this study, it was reported that Peruvian nursing professionals have a healthy level of SI in their daily practice. In addition, professional experience was identified as a predictor of SI, which can be explained by the fact that the more experienced the nurses, the greater their accumulated spiritual maturity^(15)^, integrating more moral, spiritual and ethical values to offer holistic care. They also understand that their role as caregivers gives meaning to their professional practice^(22)^. Another variable that could explain this association is age, as several studies have shown that nurses with less experience, and therefore younger, tend to have lower SI scores^(14,15)^. Along these lines, it is important to consider generational change, since millennials, who in this study represented those with less professional experience, tend to move away from traditional religious beliefs compared to previous generations, which can negatively impact their SI^(23)^.

In line with our study, Taiwanese nurses also reported that SI correlates with years of professional experience^(15)^, a finding that differs from another study carried out in China^(9)^. This discrepancy can be explained by the differences in the level of SI between the two countries, as well as the fact that nursing in Taiwan developed with fewer political restrictions than in China and with greater influence from Western medicine^(24)^. For the Chinese, spirituality is an abstract and personal concept that refers to their internal life force, the experience of suffering and the expression of their religious and cultural values^(25)^. This discrepancy suggests that, when analyzing the association between professional experience and SI, it is necessary to consider the social context, as in the case of China, which differs significantly from Latin American contexts in terms of spiritual beliefs and social cognition, influenced by macro-social processes of modernity, such as deinstitutionalization and individualization^(26)^.

The predictive relationship between religious affiliation and SI, also documented in a study carried out in China(9), shows that nurses faced challenges such as work overload and stressful situations, intensified during the COVID-19 pandemic, which generated different levels of spiritual suffering^(27)^, regardless of their religiosity^(28)^. Those who professed some religion used coping strategies such as prayer and practicing religious rituals, which may have contributed to an increase in their SI^(8)^. Although spirituality is a broader concept than religiosity, both constructs are closely related. In addition, religious practice^(29)^ and religious attitudes^(30)^ can influence the development and expression of SI, since religion is a source of cultural ideals and offers images, rituals and symbols that help people overcome adverse situations^(9)^. SI also strengthens emotional resilience in individuals experiencing stress^(31)^, allowing many to emerge emotionally strengthened during the pandemic.

In addition, the healthy level of SI reported in this study indicates that nurses have a good ability to choose conscience over ego, recognizing it as the true guide of their lives, drawing wisdom to find spiritual solutions to the problems faced in professional practice^(4)^. One explanation for this level of SI may lie in the fact that nursing professionals are characterized by their human quality, showing high levels of humanization, with behaviours such as compassion, dignified treatment, empathy, solidarity, respect, responsibility and honesty in their daily professional lives^(4)^; manifestations that were especially important during the pandemic, when the demand for humanized services was extremely necessary and indispensable.

The healthy level of SI found in Peruvian nurses is in line with a systematic review which concluded that the levels of SI reported in the primary studies analyzed were relatively high^(16)^. In addition, a study of nurses in Iran during the COVID-19 pandemic also found an acceptable level of SI^(13)^. However, this result differs from another study conducted before the pandemic on Iranian nurses, which reported a moderate level of SI^(5)^. This finding suggests that, in the face of the adversities faced by nurses during the pandemic, their SI may have increased, since this skill helps to reduce stress and exhaustion, favoring “life management” and spiritual control, allowing them to reframe their purpose and role in society^(32)^.

As for the indicators, among the three dimensions that make up SI, the one that stands out the most is Spiritual experience in practice, which involves the behavioral coherence of SI. In other words, it refers to virtuous behavior in everyday professional life. In addition, three of the six indicators in this dimension had percentages above 50%, which reveals a strong vocation on the part of the nursing staff for their mission of providing humanized care.

The factors that determine SI in nursing are not widely discussed, which makes it important to develop a line of research into the phenomenology of spirituality in order to uncover the essence of SI. This could lead to the creation of a specialty in spiritual nursing, an opportunity to broaden the understanding of the human being. Considering the scarcity of literature on SI in nursing, both in relation to practice and education, it is suggested to propose theories and models of care that address spirituality in care. In addition, it is important to promote programs to increase levels of SI in professional training, as this has a positive effect on nurses’ competence to provide spiritual care^(18)^.

### Study limitations

This research was not without its limitations. Firstly, measuring a construct such as SI, which in a way comes close to quantifying abstract or higher thoughts, can involve certain biases. However, these biases were reduced due to the use of a scale validated in a Peruvian population, built on a theoretical framework and which has high reliability. Secondly, the virtual data collection process may have generated a social desirability bias, as participants could have selected the alternatives they considered most appropriate for the role they play. To mitigate this problem, the confidentiality of information during data collection was emphasized. Finally, the choice of convenience sampling does not allow the results to be generalized to the entire nursing profession in Peru. However, in order to reduce the impact of this selection bias, a larger sample than calculated was sought, in addition to inviting nurses from different regions of Peru and from different types of healthcare establishments.

### Contributions to the field of Nursing

The association of SI with aspects related to nursing practice, such as professional experience, implies the need to expand the study of the spiritual dimension of nurses’ work, especially considering that they deal with pain and human suffering. In the coming years, it will be important to approach and interpret these phenomena from concepts derived from social and affective neuroscience, such as moral reasoning^(33)^. These conceptual approaches will make it possible to understand more clearly how religiosity, caring for others and working with human suffering activate subcortical regions related to the perception of others’ pain^(34)^.

Furthermore, based on the findings of this study, it is suggested to include the teaching of SI in undergraduate nursing programs, as this intelligence is fundamental to improving the quality of care and promoting the well-being of both patients and professionals. Scientific evidence indicates the benefits of incorporating SI into the training of health professionals, such as improved empathic communication, greater patient satisfaction and resilience to stress.

SI training can have a significant impact on the quality of care and the well-being of patients. A study carried out in Canada^(35)^ showed that SI training resulted in improved empathic communication, greater patient satisfaction and better patient-perceived quality of life. This suggests that including SI in training can contribute to more holistic and compassionate health care. In addition, teaching SI can help professionals cope with stress and burnout, increasing their resilience and decreasing levels of occupational stress^(31)^. The results of this research reinforce the importance of including SI in nurses’ training as a strategy to improve their well-being and their ability to face emotional challenges at work.

## CONCLUSIONS

The study revealed that professing a religion and length of professional experience are significant predictors of SI in the daily work context of nurses working in public and private healthcare establishments. This finding suggests that religion and the accumulation of professional experience have an important impact on nurses’ SI. As nurses gain more experience, they may develop greater spiritual maturity and ethical values that influence their practice of holistic care. In addition, religious affiliation seems to be related to higher levels of SI, possibly due to religious-based coping strategies in stressful situations. The results of this research reinforce the importance of including SI in nurses’ training as a strategy to improve their well-being and their ability to cope with emotional challenges at work.

## Data Availability

*https://doi.org/10.48331/scielodata.AVWF9Z*
