# Supplementary material for: Religion and professional experience: Are they predictors of nurses’ spiritual intelligence? Cross-sectional study
Source: Rev Bras Enferm. 2024 Dec 16;77(6):e20240217. doi: 10.1590/0034-7167-2024-0217 (PMC11654517; doi:10.1590/0034-7167-2024-0217)
Supplement: Translation

# Translation: Religión y experiencia profesional: ¿Serán predictores de la inteligencia espiritual de los enfermeros? Estudio transversal

**DOI:** 10.1590/0034-7167-2024-0217es

**Authors:** Jhon Alex Zeladita-Huaman, Juana Matilde Cuba-Sancho, Martha Brigida Martina-Chávez, Roberto Zegarra-Chapoñan, Henry Castillo-Parra

**Affiliations:** I Universidad Nacional Mayor de San Marcos. Lima, Perú; II Universidad María Auxiliadora. Lima, Perú; III Universidad San Buenaventura. Medellín, Colombia

**Keywords:** Inteligencia, Religión, Espiritualidad, Enfermeras y Enfermeros, Actividades Cotidianas.

## Abstract

**Objetivos:**

analizar la relación entre religión y experiencia profesional con la inteligencia espiritual en enfermeros.

**Métodos:**

estudio transversal y analítico realizado en 2021, participaron 544 profesionales de enfermería que laboraban en establecimiento de salud de Perú durante la pandemia por COVID-19. Para el análisis de los datos se emplearon el análisis de regresión múltiple y correlación de Pearson.

**Resultados:**

en los enfermeros predominó un nivel de inteligencia espiritual saludable (42,8%). Quienes no profesaban una religión tenían mayor probabilidad de tener menor puntaje de inteligencia espiritual (escala global y dimensiones); sin embargo, los enfermeros expertos tuvieron mayor probabilidad de tener mayor inteligencia espiritual (escala global y dimensiones) que los enfermeros novatos (p<0,05).

**Conclusiones:**

la inteligencia espiritual en los enfermeros fue predicha por la religión y la experiencia profesional. Este hallazgo sugiere que la inteligencia espiritual en enfermería se consolida mediante prácticas religiosas y durante el ejercicio profesional.

## INTRODUCCIÓN

La inteligencia espiritual (IE) nos traslada a una significación polisémica y variada. Empero, se refiere a la capacidad de la persona para buscar un sentido profundo de su existencia^(1)^; también, por esforzarse en la búsqueda de un propósito en su vida cotidiana y laboral para comprender el significado^(2)^ y la satisfacción de la vida. Incluye el nivel más alto de crecimiento en los aspectos cognitivo, ético, emocional y áreas interpersonales; que hacen que el individuo alcance plenamente la integridad interna y externa^(3)^. Con base en esos entendidos, se validaron escalas para medir al IE durante la vida cotidiana^(2)^ y en la práctica asistencial del personal de salud^(4)^. Más aún, si se tiene en cuenta que la IE está asociada con el desarrollo y transferencia de conocimiento, aprendizaje continuo, autocontrol, desempeño laboral^(5)^. Esta inteligencia es un requisito previo y componente necesario del liderazgo para lograr el desarrollo sostenible y está alineada con los Objetivos de Desarrollo Sostenible^(6)^.

En la comunidad científica, existe amplia discusión sobre la asociación de la IE con la religión. Inicialmente, cuando se definió la IE, se postuló que no necesariamente esta capacidad tiene conexión con la religión, pues ser religioso no implica tener mayor IE; pero esta inteligencia podría expresarse mediante la religión^(7)^. Sin embargo, evidencia reciente abre el debate sobre esta discrepancia. Al respecto, un estudio realizado en el Reino de Arabia Saudita encontró que los estudiantes de escuelas religiosas presentan mayor puntaje en IE; además, mediante un análisis multivariado, reportó que la religiosidad predice la IE^(8)^. En esta misma línea, otro estudio realizado en enfermeros de China concluyó que las creencias religiosas constituyen un factor predictor de la IE^(9)^.

Un estudio secundario encontró que los enfermeros con elevada IE presentan mayor competencia laboral, un sentido personal de su autocuidado fortalecido, mejor desempeño moral, excelencia personal y flexibilidad; aspectos que contribuyen a incrementar la calidad de los cuidados de enfermería^(10)^. Mientras que otra revisión sistemática reportó que la educación, la espiritualidad o creencias religiosas de las enfermeras, la edad, los años de experiencia clínica y el lugar de trabajo influyen en la comprensión de las enfermeras sobre la espiritualidad y el cuidado espiritual^(11)^. Sin embargo, no se ha encontrado estudios que analizan la relación entre las prácticas religiosas y la experiencia laboral con la IE en enfermeros latinoamericanos, a pesar de que, durante su cotidiano laboral brindan cuidado espiritual como una estrategia útil para garantizar el cuidado integral, con vocación de servicio y respetando las creencias religiosas del paciente^(12)^. Asimismo para entender las necesidades espirituales de los demás, es necesario la comprensión de la propia espiritualidad, valores, creencias^(9)^, símbolos, imágenes e imaginarios que describen la manera en que viven, es decir su cotidianidad^(13)^.

Estudios previos a la pandemia por COVID-19 reportan que los enfermeros presentan un nivel moderado de IE^(5)^; mientras que investigaciones realizadas durante la pandemia destacan un nivel aceptable^(14)^. Por otra parte, se ha reportado que esta inteligencia se encuentra asociada con características sociodemográficas y epidemiológicas como la edad, el estado civil, religión, experiencia profesional y el antecedente de haberse infectado con COVID-19^(14,15)^; así como factores relacionados a su entorno laboral como es la comunicación, percepción de la autoeficacia en el trabajo^(14)^, desempeño laboral^(5)^, el arte de enfermería, sus competencias clínicas, atributos de la práctica y compromiso personal^(16)^.

La investigación sobre la IE en los enfermeros y sus factores predictores individuales tiene implicancias para el conocimiento de la influencia que tienen las características personales en la IE durante el cotidiano laboral de enfermería, aspecto que cobra relevancia debido a que esta capacidad favorece su adaptación al entorno laboral y mejora la calidad del cuidado que brinda a los pacientes^(17)^. Asimismo, constituye un punto de partida esencial para promover el diseño de intervenciones que incrementen la IE; lo cual, facilitaría que realicen mejor su trabajo^(18)^ e incrementaría la eficiencia organizacional^(19)^. Así como, posibilitar la reflexión acerca de la importancia del abordaje espiritual en el cuidado al paciente de manera formal. Al respecto, se han encontrado insuficientes investigaciones que exploren la asociación entre la IE y las variables laborales como experiencia laboral, características sociodemográficas y profesar una religión^(5,9)^.

## OBJETIVOS

Analizar la relación entre religión y experiencia profesional con la IE en enfermeros.

## MÉTODOS

### Aspectos éticos

Esta investigación contó con la aprobación del Comité de Ética de la Universidad María Auxiliadora y el consentimiento informado de todos los participantes que se obtuvo de manera virtual. Debido a que la recolección de datos se realizó mediante un formulario electrónico, un investigador del estudio asignó un código de identificación a la información recolectada para garantizar el anonimato y proteger la identidad de los participantes, antes del análisis estadístico. Luego, el archivo que contenía el correo electrónico, único dato sensible recolectado, fue guardado en otro archivo separado de la base de datos en un servidor privado y protegido con contraseña.

### Diseño, lugar de estudio y periodo

Estudio de enfoque cuantitativo, nivel analítico y de corte transversal; realizado en la ciudad de Lima-Perú, durante los meses de abril a julio de 2021. El reporte fue guiado según STROBE.

### Población y muestra: criterios de inclusión y exclusión

Se incluyeron a profesionales de enfermería que laboraban en establecimientos de salud públicos y privados, y quienes contaban con un dispositivo electrónico (Laptop o celular) con acceso a internet para que puedan acceder al formulario virtual. Se excluyeron a profesionales de enfermería no residentes en Perú; asimismo quienes laboraban exclusivamente en el área administrativo y/o docencia. Para determinar el tamaño de muestra se consideró detectar efectos de regresión pequeños (R2=0,02) con una potencia estadística de al menos el 80% y una probabilidad de Error Tipo I de 5%, bajo estos criterios se estableció una muestra mínima de al menos 305 sujetos, sin embargo, debido a que se empleó un muestreo no probabilístico por conveniencia, para alcanzar mayor potencia estadística y reducir el impacto de sesgos individuales, se incrementó la muestra a 554 participantes. Además, se buscó la diversificación de la muestra, por lo que se invitó a participar del estudio a profesionales de enfermería que laboran en establecimientos de salud del sector público y privado de diferentes regiones de Perú.

### Protocolo de estudio

La técnica fue una encuesta virtual y como instrumento un cuestionario autoadministrado, desarrollado en *Google Forms*. La colección de datos se realizó a través de redes sociales: grupos de WhatsApp de estudiantes de la segunda especialidad de Enfermería de las universidades peruanas y Facebook de organizaciones que agrupan profesionales de Enfermería tanto a nivel gremial y de instituciones hospitalarias, igualmente, se invitó directamente a profesionales de enfermería a través de sus respectivos correos electrónicos. Los posibles factores predictores de la IE considerados fueron: el sexo, experiencia profesional (tiempo de experiencia laboral en años desde que egresó de la universidad), tipo de centro laboral; también, si realiza trabajo remoto, servicio en que labora, experiencia de haber sido diagnosticado con COVID-19. Para la variable filiación religiosa, se consideró dos categorías: religioso (si se auto definía como católico, evangélico, adventista u otro tipo de religión); no religioso (cuando el participante se consideró agnóstico o refirió que no profesaba ninguna religión).

Para la variable IE se empleó la escala de IE en la práctica sanitaria compuesta por 18 ítems^(4)^, agrupados en tres dimensiones (vivencia espiritual en la práctica, pensamiento existencial y consciencia trascendental). Cuyas alternativas de respuesta varían entre 1 a 4 puntos (1= nada verdadero para mí, 2 = algo verdadero para mí, 3= bastante verdadero para mí y 4 = totalmente verdadero para mí), respectivamente. Los niveles fueron: no saludable (<45 puntos), por mejorar (45-58 puntos) y saludable (>58 puntos).

Esta escala, inicialmente, fue diseñada para medir la IE en profesionales de la salud peruanos y mediante un estudio psicométrico con análisis factorial exploratorio determinaron que presenta validez de constructo y adecuada confiabilidad (alfa de Cronbach de 0,90)^(4)^. Posteriormente, fue empleada en otro estudio realizado en pobladores peruanos, en el cual reafirman que presenta adecuada confiabilidad (alfa de Cronbach de 0,82)^(20)^

Para efectos de este estudio, la escala de IE se validó mediante juicio de expertos; participaron tres enfermeras especialistas en salud mental y un médico con experiencia en investigación sobre IE. La calificación fue procesada según el índice de validez de contenido (CVCic)^(21)^ y se obtuvo una adecuada concordancia entre el criterio de pertinencia (CVCic = 0,944) y el criterio de claridad (CVCic = 0,935). Además, con los datos recopilados, se determinó que la escala presenta una elevada confiabilidad (coeficiente de Omega, de McDonald, de 0,93).

La probabilidad de sesgo se controló empleando instrumentos validados y confiables; con una precisión clara de los objetivos y persuasión a los participantes de que respondan el cuestionario; con una minimización de pérdidas de no respuestas; y sobre todo, cuidando el anonimato de los participantes por tratarse de un tema muy sensible.

### Análisis de los resultados y estadísticas

En función de las variables seleccionadas, la naturaleza y medición de estas como religiosidad, factores predictores e IE se empleó estadística descriptiva e inferencial, según correspondía. Se revisó el supuesto de normalidad de las variables de medición continua mediante el test de Shapiro-Willk, para confirmar el uso de pruebas paramétricas. Luego, se determinó las correlaciones de Pearson entre cada una de las dimensiones de la IE. Finalmente, se estimaron cuatro modelos de regresión para predecir el puntaje de la IE y cada una de sus dimensiones, a partir de las variables como sexo, tiempo de experiencia y religión. Para los modelos resultantes, se revisaron los supuestos de multicolinealidad, homogeneidad de varianzas y normalidad. Para considerar, que estos supuestos se cumplen se consideraron herramientas gráficas y estadísticas, como gráficos de cuantil-cuantil, un valor mayor 0,05 en la significancia del test Shapiro-Willk, el test de Levenne, y valores menores a 10 en el factor de inflación de varianza (VIF). De este modo se pudo verificar que se cumplieron con todos los supuestos satisfactoriamente. Del mismo modo, se exploró la existencia de casos atípicos; sin embargo, no se encontró ningún caso que pueda ser considerado problemático (>1) según el criterio de la D de Cook. Como criterio de significancia estadística se estableció el 0.05 como punto de corte dadas las convenciones utilizadas en la literatura existente. Todos los análisis se realizaron con el *software* R v 4.1.0.

## RESULTADOS

Participaron 544 enfermeros de 19 ciudades de Perú. La mayoría fueron mujeres (83,1 %), tenían 10 y menos años de experiencia (65,8 %), laboraba en un hospital o clínica (78,1 %), no realizaban trabajo remoto (86,8 %) y trabajaba en los servicios de Emergencia y Hospitalización (61,0 %) (Tabla 1). La edad promedio fue de 37,36 ± 10,15 años. En cuanto a la filiación religiosa, el 77,9 % (424) declaró profesar la religión católica, 13,1% (71) fueron evangélicos, 0,7% (4) adventistas y 5,7% (31) otra religión y el 2,6 % (14) se considera agnóstico o no profesa ninguna religión.

**Tabla 1:** Características de los enfermeros encuestados, 2021

Característica	n	%
Sexo		
Masculino	92	16,9
Femenino	452	83,1
Experiencia profesional (años)		
Menos de 5	210	38,6
De 5 a 10	148	27,2
De 11 a 15	80	14,7
De 16 a 20	36	6,6
Más de 20	70	12,9
Tipo de centro laboral		
Hospital o clínica	425	78,1
Centros de salud	88	16,2
Otro centro asistencial^*^	31	5,7
Realiza trabajo remoto		
No	472	86,8
Sí	72	13,2
Ha sido diagnosticado con COVID-19		
No	314	57,7
Sí	230	42,3
Servicio		
Emergencia	212	38,9
Hospitalización	120	22,1
Primer nivel de atención	76	13,9
Unidad de cuidados intensivos	55	10,2
Otro servicio†	81	14,9

* *comprende: centro de atención de emergencias, centros de atención de salud ocupacional; †comprende: servicio de neonatología, ambulancia, atención ambulatoria, entre otros.*

### Inteligencia espiritual

El 42,8 % (233) reportó una IE saludable; el 41,4 % (225), IE por mejorar y el 15,8 % (86), IE no saludable. En la Tabla 2, se describe el puntaje obtenido en la escala de IE y sus dimensiones.

**Tabla 2:** Puntaje promedio, desviación estándar, máximos y mínimos del puntaje de la escala de inteligencia espiritual y sus dimensiones en enfermeros, 2021

	Promedio ± DE	Mínimo - Máximo
Dimensión 1: Vivencia espiritual en la práctica	19,92 ± 3,38	10 - 24
Dimensión 2: Pensamiento existencial	19,80 ± 4,10	7 - 28
Dimensión 3: Consecuencia trascendental	15,10 ± 2,87	5 - 20
Inteligencia espiritual total	54,82 ± 9,54	28 - 72

En todos los indicadores de la dimensión *Vivencia espiritual en la práctica*, referida a la coherencia conductual de la IE, predomina la categoría “Totalmente verdadero para mí”, excepto en un indicador; además, en tres de ellos, el porcentaje es superior al 50 %. Mientras que, en casi todos los indicadores de las otras dos dimensiones, predomina la categoría “Bastante verdadero para mí” (Tabla 3).

**Tabla 3:** Porcentaje de los indicadores según dimensiones de inteligencia espiritual en enfermeros, 2021

Indicadores	Nada verdadero para mí	Algo verdadero para mí	Bastante verdadero para mí	Totalmente verdadero para mí
	%	%	%	%
Dimensión: Vivencia espiritual en la práctica				
2. No importa el lugar o circunstancia, siempre actúo de acuerdo a mis principios.	0,7	9,7	34,6	55,0
5. Cuando estoy abocado a la misión noble de mi práctica laboral de enfermería, mis fuerzas se multiplican.	1,5	14,7	33,3	50,6
8. Creo que cuidar mi cuerpo y el de los pacientes es un deber sagrado.	0,6	15,6	30,0	53,9
11. Cuando un paciente me necesita, siempre me hago un tiempo para ayudar.	0,4	12,1	40,1	47,4
14. Más allá del plano humano, hay un ser superior con el cual nos podemos relacionar.	2,4	17,6	41,7	38,2
17. Estoy seguro que ayudar a los demás o ser solidario con los pacientes es mi misión en esta vida.	1,1	17,3	33,8	47,8
Dimensión: Pensamiento existencial				
1. Creo que, en la vida, todo tiene un sentido profundo.	2,6	24,3	38,4	34,7
4. Cuando pienso en el milagro de mi existencia, me lleno de alegría.	5,0	17,8	32,0	45,2
7. Mi mente se apacigua cuando reflexiono sobre algún texto espiritual.	5,5	25,2	41,4	27,9
10. Frecuentemente, reflexiono sobre el significado de los acontecimientos en mi vida.	1,5	24,1	49,6	24,8
13. Soy capaz de reflexionar profundamente acerca de lo que puede haber más allá de la muerte.	7,9	31,8	39,0	21,3
16. Soy consciente que existe una conexión más profunda entre otras personas y yo.	8,1	27,6	33,8	30,5
Dimensión 3: Consciencia trascendental				
18. Es difícil para mí pensar en algo más allá que el mundo físico y material.	32,9	35,7	23,0	8,5
3. Mis momentos de práctica espiritual me renuevan las fuerzas físicas.	2,6	19,1	37,1	41,2
6. En mi tiempo libre, disfruto de la naturaleza, como jardín interior, parque, techos.	6,6	34,0	33,8	25,6
9. Cuando experimento un fracaso, todavía puedo encontrar significado en él.	2,0	29,2	46,9	21,9
12. Me defino a mí mismo por mí ser más profundo y no por mi ser físico.	0,7	18,4	49,3	31,6
15. Frecuentemente, veo las situaciones y opciones de forma más clara cuando medito, oro o rezo.	2,4	18,0	42,8	36,8

### Factores predictores de la inteligencia espiritual

El primer modelo de análisis de regresión múltiple para determinar los predictores de la IE reportó que los enfermeros que contaban con 16 y 20 años de experiencia profesional tenían 4,42 puntos más de IE; además, quienes tenían una experiencia profesional mayor a los 20 años tuvieron un puntaje de IE mayor que los participantes con menos de cinco años (p < 0,05). Por último, los enfermeros que no se consideran religiosas tenían 9,65 puntos menos de IE que aquellos que sí declaran profesar alguna religión (p < 0,001). El modelo propuesto permite explicar un 5% de la variabilidad de la IE (Tabla 4).

**Tabla 4:** Modelos de regresión para la predicción de inteligencia espiritual y sus dimensiones, 2021

	Inteligencia espiritual		Vivencia espiritual en la práctica		Pensamiento existencial		Consecuencia trascendental	
	*b*	*B*	*b*	*B*	*b*	*B*	*b*	*B*
Intercepto	54,78^*^	-	19,86^*^	-	20.11^*^	-	14.80^*^	-
Sexo (femenino)	-0,91	-0,04	-0,39	-0,04	-0,55	-0,05	0,03	0,00
Experiencia profesional (5 a 10 años)	0,39	0,02	0,15	0,02	0,20	0,02	0,04	0,01
Experiencia profesional (11 a 15 años)	2,11	0,08	1,31†	0,14	0,21	0,02	0,58	0,07
Experiencia profesional (16 a 20 años)	4,42†	0,12	1,49‡	0,11	1,60‡	0,10	1,34†	0,12
Experiencia profesional (más de 20)	2,58‡	0,09	0,98‡	0,10	0,40	0,03	1,21^**^	0,14
Profesar una religión (no religioso)	-9,65^*^	-0,16	-2,91†	-0,14	-3,80^*^	-0,15	-2,93^*^	-0,16
R^2^	0,05^*^		0,05^*^		0,03†		0,06^*^	

b - Coeficiente no estandarizado; B - Coeficiente estandarizado;

* valor de p < 0,001; †p < 0,01; ‡valor p < 0,05.

Los otros tres modelos de análisis de regresión múltiple para determinar los predictores de las dimensiones de la IE que se presenta en la tabla 4, reporta que la filiación religiosa y la experiencia profesional fueron factores predictores en todas las dimensiones. En cuanto a este último factor predictor, el puntaje de la dimensión *vivencia espiritual en la práctica* se encuentra predicha significativamente por los tres rangos de experiencia profesional considerados en este estudio; mientras que, en cuanto a la dimensión de *pensamiento existencial*, se puede observar que solamente hay diferencias entre los participantes que cuentan con un experiencia laboral menor de cinco años y aquellos con experiencia entre 16 a 20 años, donde estos últimos tienen 1,60 puntos más en esta dimensión (p < 0,05). Finalmente, para la última dimensión, *conciencia trascendental*, se observa que los enfermeros que tiene entre 16 a 20 años de experiencia tienen 1,34 puntos más (p < 0,01) y quienes tienen mayor de 20 años de experiencia presentan 1,21 puntos más (p < 0.01), que aquellos con experiencias menores de 5 años.

### Análisis de potencia estadística post-hoc

Dentro de los modelos analizados, el que tiene el tamaño de efecto más pequeño es el modelo para la predicción de pensamiento existencial; el cual, de acuerdo al R2 reportado, tiene un efecto pequeño (R2 = 0,03) equivalente a un f2 igual a 0,03. El valor para un modelo con 3 predictores, un error α de 0.05 y un tamaño de muestra total de 544 personas resulta en una potencia estadística de 0,95. Esta potencia, al ser mayor al mínimo de 0,80, indica que el estudio tuvo un tamaño muestral suficiente para los análisis presentados.

## DISCUSIÓN

En este estudio se reporta que los profesionales en enfermería peruanos presentan un nivel de IE saludable en su cotidiano laboral. Además, que la experiencia profesional fue un predictor de la IE, lo cual, podría ser explicado debido a que cuanto más expertos son los enfermeros, acumulan mayor madurez espiritual^(15)^, incorporan más valores morales, espirituales y éticos, para brindar un cuidado holístico; asimismo, entienden que su rol de cuidador le proporciona un sentido a su quehacer^(22)^. Otra variable que podría explicar esta asociación es la edad, pues diversos estudios han reportado que el enfermero con menor tiempo de experiencia laboral y por ende menor edad presenta menor puntaje de IE^(14,15)^. En esta misma línea, se debe considerar el cambio generacional, debido a que los millennials, que en este estudio estuvieron representados por quienes tenían menos experiencia profesional, suelen alejarse de las creencias religiosas tradicionales en comparación con otras generaciones anteriores, aspecto que podría tener una influencia negativa en su IE^(23)^.

Concordante con nuestro estudio, en enfermeros de Taiwán reportaron que la IE se correlaciona con los años de experiencia profesional^(15)^; hallazgo discordante con otro estudio realizado en China^(9)^. Esta discrepancia podría ser explicado porque además de que existe diferencias del nivel de IE entre ambos países señala que la enfermería en Taiwán se desarrolló con menos restricciones políticas que China y con una mayor influencia de la medicina occidental^(24)^. Asimismo, para los chinos la espiritualidad es un concepto abstracto y personal que hace referencia a su fuerza vital interna, experiencia de sufrimiento y expresión de sus valores religiosos y culturales^(25)^. Esta discrepancia sugiere que para el análisis de la asociación entre la experiencia profesional y la IE se debe considerar que contexto social, como es el caso de China que es bastante disímil de los contextos latinoamericanos en cuanto a creencias espirituales y cognición social, las cuales están influenciadas por procesos macrosocietales propios de la modernidad tales como la desinstitucionalización y la individualización^(26)^.

La relación predictiva de la filiación religiosa en la IE, que también fue documentada por un estudio realizado en China^(9)^,evidencia que los enfermeros que enfrentan desafíos como la sobrecarga laboral y situaciones estresantes, que se agudizó durante la pandemia por COVID-19, experimentaron diferentes niveles de sufrimiento espiritual^(27)^, pero que fue independiente de su religiosidad^(28)^; ante ello, quienes profesan alguna religión emplearon estrategias de afrontamiento como el rezo y realizar prácticas religiosas; lo cual podría haber conllevado a incrementar su IE^(8)^. Aunque la espiritualidad es un concepto más amplio que la religiosidad, sin embargo, ambos constructos están estrechamente relacionadas. Además, la práctica religiosa^(29)^ y las actitudes religiosas^(30)^ pueden influir en la forma en que las personas desarrollan y expresan su IE, debido a que la religión es fuente de ideales culturales y ha proporcionado imágenes, rituales y símbolos que le posibilitan vincularse a la persona con su realidad para superar situaciones adversas^(9)^. Cabe señalar que la IE además fortalece la resiliencia emocional en las personas que experimentan estrés^(31)^ que en el contexto de la pandemia por COVID-19 les permitió salir más fortalecidos emocionalmente del que entraron.

Por otra parte, el nivel saludable de IE reportado en este estudio representa que los enfermeros tienen una buena capacidad de elegir a la consciencia sobre el ego y lo reconocen como el verdadero conductor de su vida, extrayendo su sabiduría para encontrar soluciones espirituales a los problemas en el quehacer profesional^(4)^. Una explicación de este nivel de IE podría deberse a que los profesionales de enfermería se caracterizan por su calidad humana, con altos niveles de humanización, que demuestran comportamientos como compasión, trato digno, empatía, solidaridad, respeto, responsabilidad y honestidad en su cotidiano laboral^(4)^; manifestaciones importantes para un período de pandemia, en el que la demanda de servicios humanizados era sumamente necesario e imprescindible.

El nivel saludable de IE encontrado en las enfermeras peruanas corresponde con el hallazgo de una revisión sistemática en la cual concluye que los niveles de IE reportado en los estudios primarios que analizó fueron relativamente altos^(16)^, asimismo, con un estudio realizado en enfermeras de Irán durante la pandemia por COVID-19 en el cual encontraron un nivel aceptable de IE^(13)^; sin embargo, discrepa con otro estudio realizado antes de la pandemia COVID-19 en enfermeros iraníes, en el cual reportan un nivel moderado de IE^(5)^. Este hallazgo sugiere que ante las situaciones adversas que enfrentaron los enfermeros en tiempo de pandemia habría incrementado su IE debido a que esta habilidad disminuye el estrés y agotamiento; pero favorece la “gestión de la vida”, el control espiritual, que les permite resignificar su propósito y su rol en la sociedad^(32)^.

En cuanto a los indicadores, de las tres dimensiones que constituyen la IE, la que más destaca es la dimensión Vivencia espiritual en la práctica, que comprende la coherencia conductual de la IE; en otras palabras, hace referencia a los comportamientos virtuosos durante el cotidiano laboral. Aunado a que, en tres de los seis indicadores, reportan porcentajes superiores al 50 %, que estaría revelando una alta vocación del personal de enfermería con su misión de brindar un cuidado humanizado.

Los factores que determinan la IE en enfermería no está ampliamente debatido por ello que se considera importante desarrollar la línea de investigación de la fenomenología de la espiritualidad, que permita develar la esencia de la IE, lo cual posibilitaría contar con la especialidad de enfermería espiritual, que constituye una oportunidad para ampliar la comprensión del ser humano. Considerando que existe limitada literatura sobre la IE en enfermería tanto en aspectos relacionados con su práctica y en el ámbito de la educación, se sugiere proponer teorías y modelos de atención que aborden la espiritualidad en el cuidado; así como promover programas para incrementar los niveles de IE de formación profesional porque tienen efecto positivo en su competencia para proveer cuidado espiritual^(18)^.

### Limitaciones del estudio

Esta investigación no estuvo exenta de limitaciones. En primer lugar, medir un constructo como es la IE que, de alguna manera, se aproxima a cuantificar el pensamiento abstracto o superior que involucra algunos sesgos; pero se redujeron debido a que hemos empleado una escala validada en población peruana que fue construido basado en un marco teórico y presenta una elevada confiabilidad. En segundo lugar, el proceso de recolección de datos virtual podría haber generado sesgo de deseabilidad social, debido a que los participantes podrían haber seleccionado la alternativa que consideren apropiada para el rol que desempeñan. Por ello, durante la recopilación se enfatizó en la confidencialidad de la información. Finalmente, el haber optado un muestreo por conveniencia no permite que los resultados sean generalizados a todo el agremiado de enfermería peruana; sin embargo, para reducir el impacto de este sesgo de selección, se procuró un tamaño de muestra superior al calculado e invitar a participar a enfermeros de diferentes regiones de Perú que laboran en diferentes tipos de establecimientos de salud.

### Contribuciones para el área de Enfermería

La asociación que se reporta en esta investigación de la IE con aspectos relacionados con la práctica de enfermería como es la experiencia profesional, implica que para ampliar el estudio de la dimensión espiritual de la labor del enfermero, debido a que trabaja con el dolor y el sufrimiento humano, probablemente, requiera en los próximos años del abordaje y la interpretación de estos fenómenos desde conceptos derivados de la neurociencia social y afectiva como el razonamiento moral^(33)^, Además, estos acercamientos conceptuales nos permitirán entender de manera más clara y profunda cómo la religiosidad, la atención a otros y el trabajo con el sufrimiento humano pueden llevar a profesionales de la salud a activar regiones subcorticales relacionadas con la experiencias internas desde la percepción del dolor ajeno^(34)^.

Además, basado en los hallazgos de este estudio, se sugiere incluir la enseñanza de la IE en los programas curriculares de pregrado de enfermería porque esta inteligencia es de vital importancia para mejorar la calidad de la atención y promover el bienestar tanto de los pacientes como de los propios profesionales. La evidencia científica respalda los beneficios de incluir la IE en la formación de los profesionales de la salud, como la mejora en la comunicación empática, la satisfacción del paciente y la resiliencia ante el estrés.

La capacitación sobre IE a los profesionales de la salud puede tener un impacto significativo en la calidad de la atención y el bienestar de los pacientes. Según un estudio realizado en Canadá^(35)^, una capacitación en IE en profesionales de la salud condujo a una mejora en la comunicación empática, una mayor satisfacción del paciente y una mayor calidad de vida percibida por los pacientes. Estos resultados sugieren que la inclusión de la IE en la formación de los profesionales de la salud puede contribuir a un enfoque más holístico y compasivo en la atención sanitaria. Asimismo, la enseñanza de la IE puede ayudar a los profesionales de la salud a lidiar con el estrés y el agotamiento en su práctica, incluso en el aumento de la resiliencia y una disminución en los niveles de estrés laboral^(31)^. Los hallazgos de la presente investigación respaldan la importancia de incluir la IE en la formación de los enfermeros como una estrategia para mejorar su bienestar y capacidad de enfrentar desafíos emocionales propios de sus procesos de trabajo.

## CONCLUSIONES

El estudio encontró que profesar una religión y el tiempo de experiencia profesional son predictores significativos de la IE en el contexto laboral cotidiano de los enfermeros que trabajan en establecimientos de salud públicos y privados. Esta conclusión sugiere que la religión y la acumulación de experiencia profesional tienen un impacto importante en la IE de los enfermeros. A medida que los enfermeros ganan más experiencia, pueden desarrollar una mayor madurez espiritual y valores éticos que influyen en su práctica de cuidado holístico. Además, la filiación religiosa parece estar relacionada con niveles más altos de IE, lo que podría atribuirse a estrategias de afrontamiento basadas en la religión utilizadas en situaciones estresantes. Los hallazgos de la presente investigación respaldan la importancia de incluir la IE en la formación de los enfermeros como una estrategia para mejorar su bienestar y capacidad de enfrentar desafíos emocionales propios de sus procesos de trabajo.

## DISPONIBILIDAD DE DATOS Y MATERIAL

https://doi.org/10.48331/scielodata.AVWF9Z
